# Image-Based Detection and Classification of Malaria Parasites and Leukocytes with Quality Assessment of Romanowsky-Stained Blood Smears

**DOI:** 10.3390/s25020390

**Published:** 2025-01-10

**Authors:** Jhonathan Sora-Cardenas, Wendy M. Fong-Amaris, Cesar A. Salazar-Centeno, Alejandro Castañeda, Oscar D. Martínez-Bernal, Daniel R. Suárez, Carol Martínez

**Affiliations:** 1 Faculty of Engineering, Pontificia Universidad Javeriana, Bogotá 110311, Colombia; j_sora@javeriana.edu.co (J.S.-C.); wendy.amaris@icb.ufpa.br (W.M.F.-A.);; 2 Programa de Doutorado em Biotecnologia, Universidade Federal do Pará, Belém 66075-110, Brazil; 3 Computer Vision Lab, Delft University of Technology, 2628 XE Delft, The Netherlands; 4 Space Robotics Research Group (SpaceR), Interdisciplinary Centre for Security, Reliability and Trust (SnT), University of Luxembourg, L-1855 Luxembourg, Luxembourg; carol.martinezluna@uni.lu

**Keywords:** malaria diagnosis, thick blood smears, image processing, support vector machines, convolutional neural networks, deep learning

## Abstract

Malaria remains a global health concern, with 249 million cases and 608,000 deaths being reported by the WHO in 2022. Traditional diagnostic methods often struggle with inconsistent stain quality, lighting variations, and limited resources in endemic regions, making manual detection time-intensive and error-prone. This study introduces an automated system for analyzing Romanowsky-stained thick blood smears, focusing on image quality evaluation, leukocyte detection, and malaria parasite classification. Using a dataset of 1000 clinically diagnosed images, we applied feature extraction techniques, including histogram bins and texture analysis with the gray level co-occurrence matrix (GLCM), alongside support vector machines (SVMs), for image quality assessment. Leukocyte detection employed optimal thresholding segmentation utility (OTSU) thresholding, binary masking, and erosion, followed by the connected components algorithm. Parasite detection used high-intensity region selection and adaptive bounding boxes, followed by a custom convolutional neural network (CNN) for candidate identification. A second CNN classified parasites into trophozoites, schizonts, and gametocytes. The system achieved an F1-score of 95% for image quality evaluation, 88.92% for leukocyte detection, and 82.10% for parasite detection. The F1-score—a metric balancing precision (correctly identified positives) and recall (correctly detected instances out of actual positives)—is especially valuable for assessing models on imbalanced datasets. In parasite stage classification, CNN achieved F1-scores of 85% for trophozoites, 88% for schizonts, and 83% for gametocytes. This study introduces a robust and scalable automated system that addresses critical challenges in malaria diagnosis by integrating advanced image quality assessment and deep learning techniques for parasite detection and classification. This system’s adaptability to low-resource settings underscores its potential to improve malaria diagnostics globally.

## 1. Introduction

Malaria remains a significant global health concern, particularly affecting low-income countries where resources are scarce and healthcare systems are overburdened. According to the World Health Organization (WHO), an estimated 249 million malaria cases occurred in 2022, resulting in approximately 608,000 deaths worldwide [1]. The disease is caused by the *Plasmodium* parasite and is transmitted to humans through the bite of the female *Anopheles* mosquito. Among the various species, *Plasmodium falciparum* is the most prevalent and lethal [2].

The current gold standard for malaria diagnosis involves manually counting parasites in stained blood smears—a labor-intensive and highly subjective process [3,4]. This method requires well-trained microscopists, which makes it impractical in many endemic regions due to a shortage of skilled personnel [5]. Two primary methodologies are employed: thin blood smears, which provide a single layer of red and white blood cells (WBCs), and thick blood smears, which concentrate multiple layers of blood for higher sensitivity [6]. While thick smears enhance parasite detection, they present challenges such as overlapping cells and staining artifacts that complicate analysis. Standard staining methods include Giemsa and Romanowsky dyes, with the latter being preferred for its stability in humid climates [5]. Maintaining staining quality is essential for accurate parasite visualization, yet it remains problematic in resource-limited settings.

Manual microscopic examination for parasite detection, life stage differentiation, and parasite counting is laborious and subjective [5]. Additionally, the WHO acknowledges that microscopists often work in low-resource and isolated environments without systems to ensure diagnostic quality. Heavy workloads and a lack of trained health personnel further limit the effectiveness of microscopy in regions with a high disease burden [5]. These challenges have prompted the development of computational image-processing methods to support malaria diagnosis. Such tools improve the reliability of test interpretations, reduce healthcare workers’ workload, and lower diagnostic costs [6].

Existing computational methods primarily focus on parasite detection but often fail to address other critical aspects, such as smear quality assessment and leukocyte detection, leaving a significant gap in comprehensive diagnostic solutions. Various machine learning (ML) approaches have been employed to detect and quantify parasites in stained blood smear images. For example, Rosado et al. [7] developed a method to detect *Plasmodium falciparum* trophozoites and WBCs in Giemsa-stained thick blood smears, achieving 80.5% recall, 93.8% specificity, and 91.8% accuracy at the patch level using adaptive thresholding and a support vector machine (SVM) classifier. Dave et al. [8] applied histogram-based adaptive thresholding and mathematical morphological operations for segmentation, achieving 86.34% recall and 96.60% specificity at the patch level with a cubic SVM for classifying parasites in different life stages. Delahunt et al. [9] described an automated malaria diagnosis system for thick smears, achieving 95% specificity at the patient level using morphological, color, and texture features with a linear SVM. However, classifying parasite stages in thick blood smears remains particularly challenging, with limited studies reporting accuracies of around 76%. Furthermore, many existing methods require high computational resources and extended processing times, which are impractical in resource-limited environments.

Deep learning (DL) techniques have emerged as powerful tools for automatic feature extraction and detection in thick blood smears. Quinn et al. [10] proposed a convolutional neural network (CNN) model for parasite detection, reporting an average precision of 97% using smartphone-captured images divided into patches. Mehanian et al. [11] utilized CNN models for parasite detection and quantification, achieving recall, precision, and specificity of 91.6%, 89.7%, and 94.1%, respectively, although with a processing time of 20 min. Yang et al. [12] introduced smartphone-based algorithms employing customized CNN and Faster R-CNN models for parasite detection, achieving detection rates of 96.84% and 96.81% at the image and patient levels, respectively. Despite these advancements, challenges such as classifying parasite stages in thick blood smears and evaluating staining quality for parasite visualization still need to be explored. In our previous work, we developed an image-based approach using the HSV color space and an SVM, achieving an F1-score of 97% for classifying smear quality [13]. However, the detection of leukocytes and assessment of staining quality still need to be explored.

Our research addresses these critical gaps by developing an integrated system that detects and classifies malaria parasites, assesses image quality, and counts leukocytes in Romanowsky-stained thick blood smears. This novel and comprehensive approach can transform malaria diagnostics by providing a cost-effective and scalable solution accessible to remote and resource-limited areas. Moreover, its ease of integration into existing diagnostic workflows ensures that minimal additional training is required for healthcare personnel.

In summary, this paper proposes a comprehensive system that (1) automatically assesses image quality, (2) detects and counts leukocytes, and (3) detects and classifies malaria parasites in images of Romanowsky-stained thick blood smears, a diagnostic medium often overlooked despite its relevance in tropical regions. Our approach fills gaps in malaria diagnostics by leveraging image processing, classical ML, and DL techniques. Unlike previous studies focusing solely on parasite detection, our system offers a holistic diagnostic tool for resource-limited settings. By integrating these components, we aim to improve diagnostic accuracy, facilitate prompt treatment, and ultimately contribute to reducing the global burden of malaria.

## 2. Materials and Methods

This section outlines the methodology for quality analysis, leukocyte detection, and malaria parasite detection and classification in Romanowsky-stained thick blood smear images of *Plasmodium vivax*. This study analyzed 1000 anonymized images previously used for actual diagnoses and labeled them with information on color quality, diagnosis, leukocyte location, parasite count, location, and stage.

**Figure 1:**
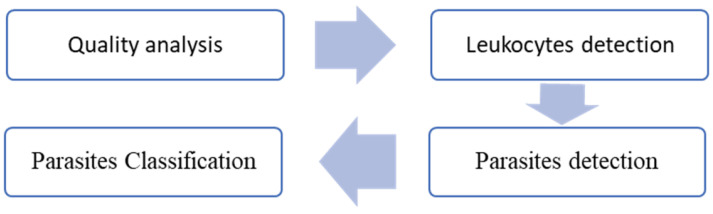
Block diagram of the proposed method.

Image quality was assessed using feature extraction techniques based on the HSV color space and support vector machines (SVMs). Leukocyte detection involved experimental designs leveraging distinct color spaces and image enhancement techniques. Parasite detection was performed by classifying parasite candidates (cropped image segments) using a custom convolutional neural network (CNN). The SVM and CNN methods were further compared to classify parasite stages, as illustrated in Figure 1.

### 2.1. Image Dataset

**Figure 2:**
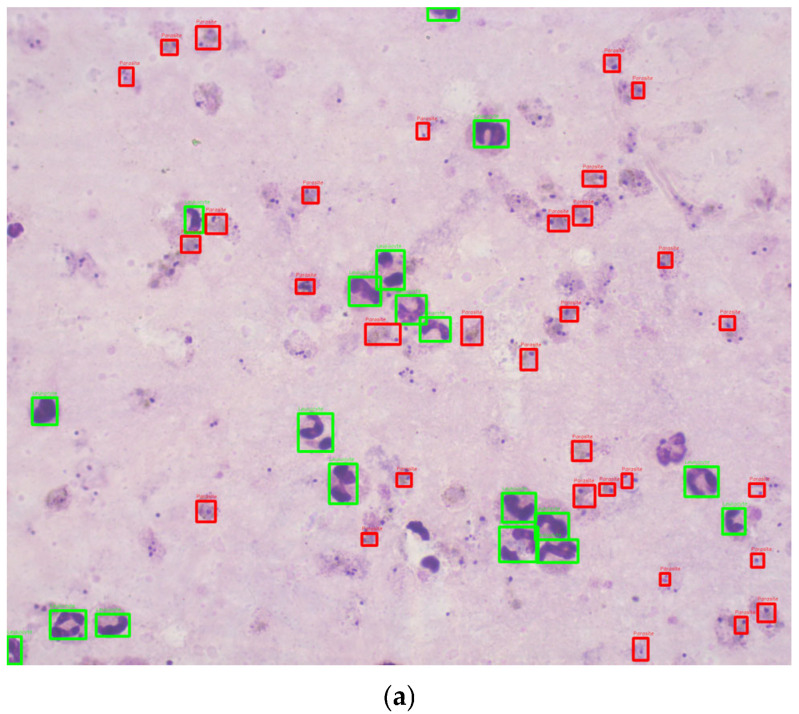

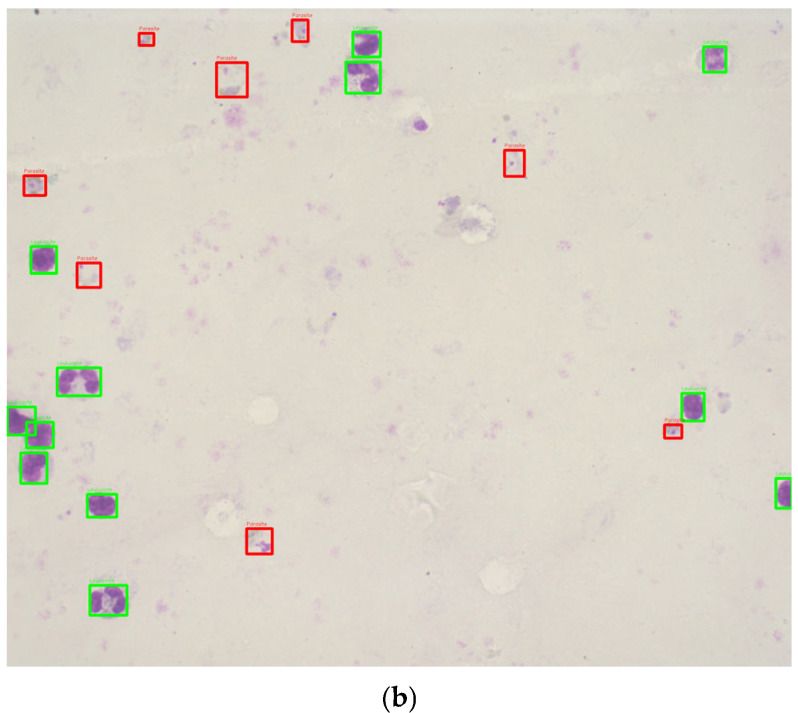
Sample images annotated with red (parasite) and green (leukocytes) bounding boxes. (**a**) Good quality image sample. (**b**) Bad quality image sample.

This study utilized 1000 Romanowsky-stained thick blood smear images from the National Institute of Health in Colombia. These images were derived from 100 slides, with ten images captured per slide near the sample center to ensure uniformity. Each image was taken with a Zeiss Scope A1 optical microscope at 100× magnification, resulting in RGB color images with a resolution of 2452 × 2056 pixels. The images were annotated by experts, using bounding boxes to label the life stages of each parasite (Figure 2). To the best of our knowledge, no previous studies have used datasets of Romanowsky-stained thick blood smears, a dye commonly employed in tropical climates due to its stability.

The database expands on a previous dataset of 420 images created by one of the authors, used initially to evaluate the coloration quality of thick blood smears (TBS) [13]. For this new project, the same methodology from the prior work was applied to collect and annotate an additional 580 images, resulting in 1000 images.

The images were captured using a standardized methodology to ensure consistency in color representation and image quality:

- The dataset was created using a 100× magnification Axio Zeiss Scope A1 optical microscope (Carl Zeiss, Oberkochen, Germany). The LED-illuminated microscope eliminated the need for a blue filter.
- Key microscope components, such as the reflector insert, field diaphragm, and aperture diaphragm, were kept in fixed positions to standardize the lighting conditions. As malaria diagnosis professionals at the National Health Institute of Colombia (INS) recommended, the light intensity was calibrated at 22.4 lux using a light meter (Model 407026, Extech, Nashua, NH, USA).
- The images, each with a resolution of 2056 × 2452 pixels, were captured in PNG format and stored with annotations of relevant biological features.

The original 42 thick blood smear samples were collected from malaria cases caused by *Plasmodium vivax* during 2017–2018 [13]. Each thick blood smear was photographed in 10 central fields to ensure uniformity and avoid variability caused by peripheral blood thickness. This methodology was replicated for additional slides to expand the dataset.

The images were annotated by personnel certified in malaria parasite stage identification using the web-based tool Labelbox [14]. The annotations include bounding boxes identifying parasite life stages (e.g., trophozoites, schizonts, gametocytes) and leukocyte locations.

#### Data Partitioning

The dataset consisted of 1000 Romanowsky-stained thick blood smear images, including 217 classified as good and 783 as bad quality. Among these, 702 images contained parasites, while 298 did not. A total of 6188 parasites were annotated, distributed as 5927 trophozoites, 114 schizonts, and 147 gametocytes, with sizes ranging from 13 to 138 pixels. Additionally, 12,712 leukocytes were identified, ranging in size from 15 to 222 pixels. Twelve images lacked leukocytes, while others contained up to 43, with an average of 13 leukocytes per image.

The images were annotated by personnel certified in identifying malaria parasites, including one of the authors, who also contributed to the previous work on the original dataset [13]. The classification into “good” and “bad” quality was based on criteria defined by the National Health Institute of Colombia (INS) and aligned with WHO protocols [15], focusing on background coloration and staining quality.

The distribution of a higher proportion of low-quality images than high-quality ones reflects the real-world conditions in resource-limited settings where malaria diagnoses are commonly made. Low-quality images are more prevalent in these settings due to available equipment and technology constraints. Prioritizing low-quality images in the training dataset enables the model to develop greater resilience and effectiveness when processing suboptimal images, ultimately improving its performance in actual field conditions. By incorporating a more significant number of low-quality images, the model’s ability to accurately identify and classify malaria cases is enhanced, even when image quality is less than ideal, a situation often encountered in rural laboratories or areas with limited resources. This strategy also addresses the challenge posed by the variability in image quality, a common issue in malaria diagnosis in settings with limited technical resources [16].

The dataset was divided into training (70%), validation (15%), and testing (15%) subsets using a stratified sampling approach. This split was conducted separately for good and bad quality images, ensuring the same distribution of quality categories in each subset. Although the dataset contained more bad quality images than good quality images, balancing adjustments were not performed during partitioning. Instead, class balancing was addressed during the detection and classification phases.

**Figure 3:**
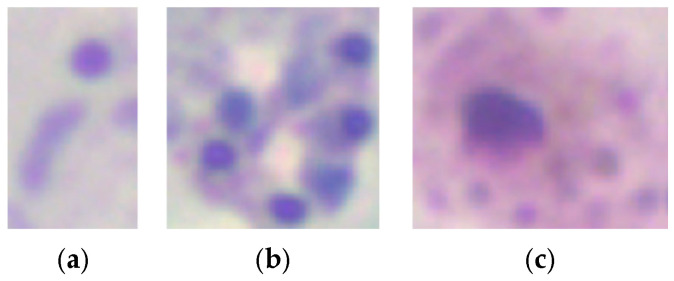
Example crops for each parasite stage: (**a**) trophozoites, (**b**) schizonts, (**c**) gametocytes, with sizes ranging from 13 to 138 pixels (100×).

Data augmentation was applied to mitigate class imbalance during model training. For parasite detection, augmentation techniques such as rotations, horizontal and vertical flips, and combinations of flips were applied at the patch level, increasing the dataset size. Similarly, data augmentation was applied to underrepresented classes (schizonts and gametocytes) for parasite stage classification, while subsampling was performed on the trophozoite class. Figure 3 shows example crops for each parasite stage: (a) trophozoites, (b) schizonts, and (c) gametocytes.

The dataset was split into training, validation, and testing subsets as a hold-out validation approach. Table 1 summarizes the distribution of the dataset after partitioning.

### 2.2. Image Quality Analysis

In the literature, red, green, and blue (RGB) have been the color space most frequently used for malaria diagnosis [5,12,17,18]. Furthermore, hue, saturation, and brightness (HSV) have been used for malaria parasite detection [5,19,20]. This methodology builds upon prior work by Fong et al. [13], incorporating HSV-based feature extraction and SVM classification to improve image quality assessment. This integration represents an advancement in automating diagnostic processes tailored to Romanowsky-stained thick blood smears. The authors also indicated that background thresholding allowed the separation of foreground elements (leukocytes, platelets, parasites) from the background, explicitly using the H and S components of the HSV color space. Alternatively, the data distribution in the RGB color space did not differentiate the two background quality classes. After visually analyzing the histograms, the usefulness of the H and S components in the HSV color space was confirmed. Therefore, the H and S components were used in an HSV histogram to remove the leukocytes and parasites (foreground elements). Then, the threshold image was applied to the original image as a mask, resulting in an image that retained the background information in the HSV color space, with the foreground elements in black.

Leukocyte and parasite detection were chosen as parameters for assessing image quality due to their importance in ensuring the reliability of malaria diagnoses. Poor staining directly affects the ability to identify these elements, leading to faint or distorted appearances. Leukocytes are particularly valuable for evaluating staining consistency and intensity as they are often the most evident elements in well-stained smears. Parasites provided additional insight by assessing the visibility of their stages (trophozoites, schizonts, gametocytes) and the differentiation from artifacts. These parameters align with established guidelines and previous work [13].

Subsequently, the SVM method was employed to classify the images into good and bad quality categories using histogram bins for feature extraction. The H and S components from the HSV space were used to create histograms with 16 bins, removing the first bin to exclude noise from foreground elements. This process allowed for the identification of optimal features for quality classification.

### 2.3. Leukocyte Detection

**Figure 4:**
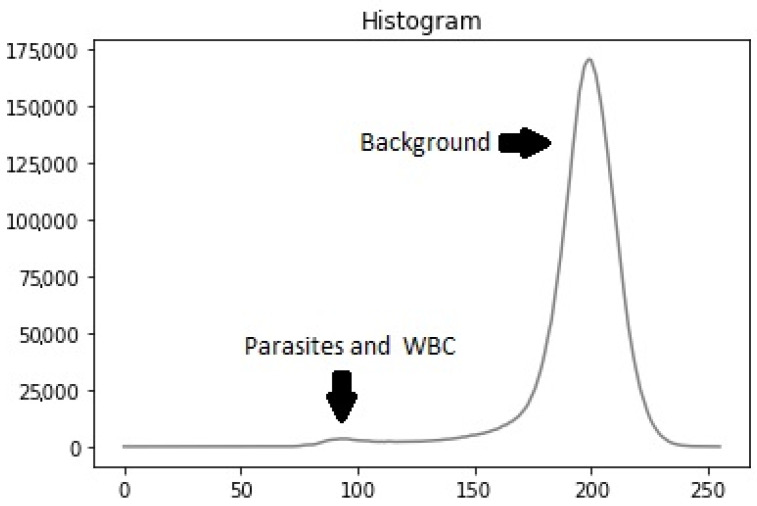
Histogram example.

Previous studies have used various image-processing techniques for leukocyte detection and counting, typically involving a preprocessing stage followed by segmentation. These methods include contrast stretching and adaptive thresholding on channel V [20], low-pass filtering with contrast stretching and optimal thresholding segmentation utility (OTSU) [21], and Gaussian low-pass filtering with adaptive histogram equalization and adaptive thresholding [22]. Our dataset’s histogram analysis revealed that particles (leukocytes, parasites, platelets) had low intensity while the background had high intensity, resulting in a bi-modal intensity distribution (Figure 4). To address this, we used the OTSU method [23] to generate a binary mask for segmenting stained particles and detecting white blood cells (WBCs), removing the background to simplify the computational process.

**Figure 5:**
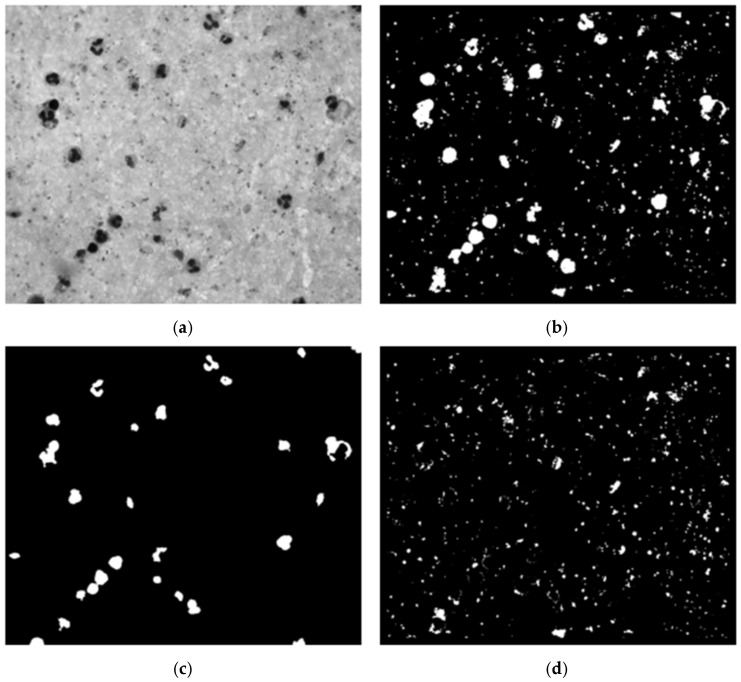
Example of WBC segmentation process. (**a**) Grayscale image; (**b**) OTSU’s segmentation; (**c**) erosion function; (**d**) mask candidates.

We conducted an experimental design to optimize preprocessing for effective leukocyte segmentation. Various color spaces were evaluated, including grayscale, channels R, G, and B from RGB, and channels S and V from HSV. The filters assessed included low-pass, Gaussian low-pass, median filter, and no filter. The contrast enhancement techniques evaluated were contrast-limited adaptive histogram equalization (CLAHE), contrast stretching, and no contrast enhancement. Using OTSU’s method, a binary mask was generated, followed by noise removal with an erosion function to eliminate small white noises (platelets and parasites) (Figure 5c). Finally, the connected components algorithm was applied to develop a leukocyte detection and counting algorithm.

Building upon earlier studies focused on thin blood smears, we documented the use of machine learning for leukocyte detection in thick blood smears. The features utilized included geometric [20,24,25,26,27], statistical [20], textural [20,28], intensity [28], and spectrum-based features [29]. The gray level co-occurrence matrix (GLCM) was used to extract statistical and textural features, while pixel intensity was used to extract intensity features. Three feature groups were created: one with four variations for each GLCM feature, another with twelve variations, and a third consisting of the pixel intensity of a 50 × 50 pixels size image (2500 features vector). Classic machine learning algorithms, including naive Bayes, decision trees, support vector machines (SVM), and k-nearest neighbors (k-NNs), were employed to classify leukocyte candidates as either leukocyte or noise.

### 2.4. Parasite Detection

We began parasite detection by selecting candidates from high-intensity regions within the image. These candidates were used to train a custom convolutional neural network (CNN) to distinguish parasites from background elements. This approach was computationally efficient as it reduced the data processing size compared with the original image, as outlined by Feng et al. [30]. Unlike the SVM approach for image quality assessment, CNNs were more suitable for complex tasks such as feature extraction and classification, both essential for parasite detection.

Variations in staining or illumination during blood smear preparation can cause segmentation and classification challenges [31]. To address these challenges, we optimized preprocessing using an experimental design similar to that for detecting WBCs, minimizing parasite exclusion during segmentation (Figure 5b). The parameters used were based on prior malaria parasite detection methods to ensure consistency, as documented in review articles [22,32].

Using the WBC mask from the previous step, we removed leukocytes from the segmentation mask, retaining candidates predominantly corresponding to parasites, platelets, and some background noise. The segmentation process, illustrated in Figure 5, included identifying each image segment’s center coordinates. An adaptive bounding box was created around these coordinates, starting at 40 × 40 pixels and dynamically expanding to encompass the entire candidate region until no white pixels were found along the edges. The maximum size of the bounding box was capped at 90 × 90 pixels. Once the bounding box was appropriately sized and positioned, the image was cropped based on the identified contours, retaining only the relevant portion containing the parasite candidate for further analysis.

The model’s performance was evaluated using accuracy, precision, recall, and F1-score. These metrics were calculated at multiple stages: image quality assessment, leukocyte detection, and parasite classification. The F1-score was chosen as the primary metric due to its robustness in handling imbalanced datasets. At the same time, precision and recall highlighted the model’s ability to minimize false positives and false negatives.

#### Training

The model training process involved using the images of parasites within bounding boxes provided by experts. There were 4364 parasites. These parasites were removed from the candidate mask for images with parasites, and the resulting clippings were obtained. Due to class imbalance, data augmentation was applied to the parasite images, including rotations of 90 and 180 degrees, horizontal and vertical flips, and combinations of flips, resulting in 26,184 images. Additionally, subsampling was performed on the non-parasite class to balance the training data.

The CNN model architecture comprised eleven convolutional layers and five max-pooling layers interspersed between each convolutional layer. A normalization batch layer was utilized to facilitate a higher learning rate [33], followed by rectified linear units (ReLUs) as the activation function [34]. Subsequently, three fully connected layers were included, with 1024, 512, and 2 hidden units, respectively, followed by a SoftMax layer. Two dropout layers with a dropout ratio of 0.5 were inserted between the fully connected layers to mitigate overfitting [35]. The output of the CNN model was a vector indicating the likelihood of the input image patch being a parasite or non-parasite.

Furthermore, pre-trained networks such as VGG-19 [36], MobileNetV2 [37], and ResNet-50 [38] were utilized to compare the developed model’s performance.

### 2.5. Parasite Stage Classification

The CN For the SVM model design, features extracted from histograms in the red and saturation channels were found to be more variable across different parasite stages. Sixteen bins were chosen for each histogram to reduce the dimensionality of the feature vector. Additionally, the number of nuclei was included as a feature, particularly since it exhibits significant variation in schizonts. The presence of parasites and coloration quality were also considered during feature selection. A factorial experiment was conducted to determine the optimal SVM classifier based on factors such as kernel type, gamma, and learning ratio.

Subsequently, various neural network configurations were evaluated for CNN. Initially, different image sizes were evaluated, including [25 × 25], [50 × 50], [100 × 100], and [150 × 150]. After identifying the most suitable layer and convolution distribution, a factorial experiment was conducted, varying parameters such as image scale, learning ratio (ranging from 1 × 10^−4^ to 1), batch size (ranging from 20 to 100), and the number of epochs (ranging from 50 to 150). Multiple networks were implemented, including VGG-16, MobileNetV2, and ResNet-50 architectures, with fixed batch sizes of either 50 or 100 and a number of epochs set to either 50 or 100. No frozen layers were utilized for these networks, and an image size of [50 × 50] was employed for evaluation. The evaluation of these models aimed to determine the best-performing network for classifying parasite stages based on their size, shape, and internal structures.

## 3. Results and Discussion

### 3.1. Image Quality Analysis Performance

Table 2 shows the implementation results of quality analysis performance. The cubic kernel has the best performance, with a precision of 95%, an F1-score of 95%, an accuracy of 95%, a true negative rate of 96%, and a true positive rate of 93%. These results suggest that the system is effective at predicting the negative class, which has positive implications as it allows for generating a better warning number and provides more control over the cases.

The methodology employed in this study closely follows that of Fong Amaris et al. [13], which proposes automating coloration quality estimation in thick blood smears (TBSs). This technique has not been deeply explored before, making it a novel contribution to malaria diagnosis. Given that the accurate assessment of image quality is crucial for automated TBS analysis, which has not been widely studied, this study focuses on a new approach that enhances diagnostic accuracy by integrating image quality evaluation directly into the process.

### 3.2. Leukocyte Detection Performance

#### 3.2.1. Image Processing

In the experimental design, seventy-two masks were used to detect leukocytes (Table 3). The best results were obtained using the blue channel, no filter, and CLAHE for contrast enhancement, followed by optimal thresholding segmentation utility (OTSU) segmentation and connected components.

Using the testing set, the blue channel method (no filter with CLAHE using OTSU segmentation followed by mask noise removal and connected components) yielded an average precision of 86.27%, recall of 93.82%, and F1-score of 88.52%.

#### 3.2.2. Machine Learning

The results showed (Table 4) that the best combination for the leukocyte detection mask using Romanowsky dye involved the blue channel, with no filter, employing CLAHE for contrast enhancement, followed by OTSU segmentation, mask noise removal, and the connected components algorithm. This combination applied to the testing set resulted in an average precision of 86.27%, an F1-score of 88.52%, and an accuracy of 88.52%.

Different image processing techniques exist for detecting and counting leukocytes, including several techniques using various color spaces such as grayscale [12,21,22,39] and HSV [8,20,22]. However, neither the RGB color space nor its channels have been used. Therefore, the experimental design implemented each color space as a variable. Additionally, different image preprocessing techniques, such as filtering, and contrast enhancement methods, such as contrast stretching [20], low-pass filter with contrast stretching [18], Gaussian low-pass filter with adaptive histogram equalization [22], and low-pass filter with contrast stretching [22, 23], were tested using a specific color space; however, not all possible combinations were implemented. According to our experimental design, the best combination of filtering and contrast enhancement for the detection leukocytes mask was using no filter with contrast-limited adaptive histogram equalization (CLAHE) on the blue channel, obtaining an average precision, recall, and F1-score of 86.26%, 93.82%, and 88.52%, respectively, using the test set.

There is no documentation about implementing machine learning to detect and count leukocytes in thick blood smears. For that reason, different features like statistical features [26] and textural features [27,28] were analyzed using the gray level co-occurrence matrix (GLCM) and intensity features [27] using the pixel intensity of the image, all of which were used for classification. As a result, the support vector machine (SVM) algorithm distinguished between leukocyte and noise in terms of accuracy (88.92%), precision (89.36%), sensibility (88.92%), specificity (89.95%), and F1-score (88.69%). Compared with the machine learning results in the previous phase (image processing), it is possible to notice that the increased performance between both phases is minimal compared with the computational resources and speed of the entire algorithm, with a 3.1% precision and a 0.16% F1-score.

While prior works like Quinn et al. [10] have explored CNN-based approaches for leukocyte detection, these methods often require more computational resources. Our approach demonstrates that traditional machine learning algorithms remain competitive, particularly in resource-limited settings.

### 3.3. Parasite Detection Performance

#### 3.3.1. Performance of Candidate Identification

The algorithm’s performance in detecting parasite candidates was evaluated, considering that a parasite is correctly identified if the center of the annotation created by the expert is between a radius of thirty-five pixels to the center of the contour obtained with the segmentation of the algorithm. A radius of thirty-five pixels was selected by varying its values between 20 and 120 pixels and selecting the radius that identified the highest number of parasites. Subsequently, the algorithm’s recall was evaluated at the image level, the slides’ level, and the entire dataset (the relationship between the number of indeed identified parasites and the total number of annotated parasites). The results achieved a recall of 93.29% at the image level and 93.35% at the slide level.

The proposed IGMS by Yang et al. [12] achieves a recall of 97.49% ± 5.40% on an image level and 96.59% ± 5.52% on a patient level, respectively. They used Giemsa-stained thick blood smear slides from 150 *P. Falciparum*; however, our model used both bad and good quality Romanowsky-stained images, and the bounding boxes did not have a standard size.

#### 3.3.2. CNN Model

**Figure 6:**
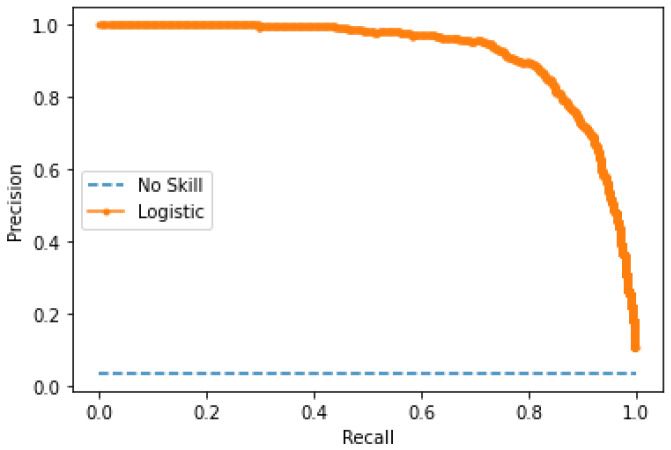
Precision curve of the customized CNN model on patch level.

The performance of the custom CNN model was evaluated using the test dataset, which consists of fifteen slides for a total of 150 images, 543 parasites, and 9.580 background noise. Our model achieved an accuracy of 98.65%, an F1-score of 82.10%, a specificity of 99.10%, sensitivity of 86.55%, and precision of 78.07%, a false positive rate of 0.90%, and a false negative rate of 13.44%. The precision curve was also calculated as it is more informative when evaluating classifiers with an unbalanced dataset in an ROC graph [40]. The AUC was 0.912, showing the effectiveness of the CNN model. The corresponding precision curve and confusion matrix are shown in Figure 6 and Table 5 and Table 6.

Yang et al. [12] obtained the following performance metrics on patch level: accuracy 97.26%, AUC 97.34%, recall 82.73%, specificity 98.39%, precision 78.98%, and F1-score 80.81%. These results are similar to those obtained with our customized CNN model, demonstrating consistent performance across different approaches.

Quinn et al. [10] proposed a convolutional neural network (CNN) model for parasite detection, reporting an average precision of 97% using smartphone-captured images divided into patches. Their approach achieved impressive precision, indicating the potential of mobile-based systems for malaria detection. Our results align with theirs, but we also focus on improving the robustness of our model in dealing with image quality variations.

Mehanian et al. [11] utilized CNN models for parasite detection and quantification, achieving recall, precision, and specificity of 91.6%, 89.7%, and 94.1%, respectively, although their method took 20 min to process. While their model showed strong performance, our model offers a more efficient solution with faster processing times, further enhancing its practicality for real-world use.

Rahman et al. [18] proposed a deep learning model for malaria detection in red blood cell smears, achieving an accuracy of 97.77% using a deep convolutional neural network (CNN). Their method differs from ours in that they directly use raw segmented red blood smear patches, avoiding hand-engineered feature extraction. While Rahman et al. achieved very high accuracy with a dataset from the NIH malaria dataset, their model was trained and tested using preprocessed images. In contrast, our approach accounts for non-ideal images, offering a more robust solution in varied real-world conditions.

Kaewkamnerd et al. [19] focused on parasite detection and classification using thick blood films, reporting a classification success rate of 90% for *Plasmodium falciparum* (Pf) and 75% for *Plasmodium vivax* (Pv). Their work highlights the importance of thick blood films in detecting parasitic presence, especially when parasite concentration is low in thin films. Our results in parasite detection align with their findings, supporting the use of thick blood films in malaria diagnosis.

### 3.4. Parasite Stage Classification Results 

#### 3.4.1. Machine Learning SVM Model

For the model’s design based on SVM, changing the gamma and learning rate hyperparameters did not affect the model’s performance. Therefore, only the kernel was analyzed. Accuracies of 65%, 52%, and 54% were obtained for the linear, quadratic, and cubic kernels. Furthermore, the system was more effective in all models when classifying the schizonts class, followed by the trophozoites class, as shown in Table 6.

#### 3.4.2. Deep Learning

When conducting a preliminary analysis, it was found that the best composition consisted of three convolutional layers and two hidden layers of the neural network with 512 and 256 neurons, respectively, with an accuracy of 84%. After performing hyperparameter tuning, 87% accuracy was reached using the test set. The trophozoites class was the best classified in this model, followed by the schizonts class. Table 7 shows the performance of the four best results of the experiments for multiple configurations with the CNN model.

Afterward, the best configuration was used, and hyperparameters were adjusted to improve the network’s performance. Table 8 shows the performance by varying hyperparameters.

Finally, multiple networks (Table 9) were implemented with default values without frozen layers. The architectures that were evaluated were VGG-16 [36], MobileNetV2 [37], and ResNet-50 [38]. They had a fixed batch size [50; 100] and number of epochs [50; 100]. No frozen layers were used for these networks, and an image size of [50 × 50] was used.

Identifying some descriptive characteristics that allowed the different stages to be classified was possible. Various characteristics that do not present graphically separable patterns were also explored. It was also possible to conduct a good class balance approach and reduce the differences with trophozoites. When multiple values were applied to the gamma and C parameters of the classifier, it was evident that they did not represent a change in the classifier’s performance, so its default value was left. Also, in all tests, the linear classifier had the highest performance.

Dave et al. [8] used Giemsa-stained images to classify all parasite stages (*ring*, *trophozoite*, *schizont*, and *gametocyte*) in thick blood smears. Unlike other methods that only classify the *ring* stage, their system can classify the complete parasite lifecycle, which is crucial for accurate diagnosis and treatment.

The algorithm showed a 7.14% discrepancy compared with expert microscopists, indicating reliable performance. Additionally, the system can estimate parasite density, providing valuable information for malaria severity assessment.

While this study is one of the few to address stage classification in thick blood smears, it highlights a significant gap in the field, as parasite stage classification in thick blood smears is an opportunity for further research.

## 4. Conclusions

In this paper, we presented an automated system for assessing image quality, detecting leukocytes, and detecting and classifying malaria parasites in Romanowsky-stained thick blood smears of Plasmodium vivax. The proposed pipeline integrates feature extraction techniques based on HSV color space and support vector machines (SVMs) for image quality assessment and a custom convolutional neural network (CNN) for parasite detection and classification. These components collectively address key aspects of the malaria diagnostic process.

This study introduces a novel approach by automating image quality estimation and parasite stage classification, areas that have been insufficiently explored in previous research. Integrating image quality analysis in the diagnostic pipeline significantly enhances the accuracy and reliability of malaria detection, even in cases where images may have varied quality.

The results indicate that the SVM achieved a precision of 95% for image quality assessment, while the leukocyte detection yielded an accuracy of 88.92%. For malaria parasite detection, the custom CNN model attained an accuracy of 98.65% and effectively classified parasite stages, with F1-scores of 85% for trophozoites, 88% for schizonts, and 83% for gametocytes.

These findings demonstrate the effectiveness of our system in automating various stages of the malaria diagnostic process, even when working with images of heterogeneous quality. The balance between accuracy and computational efficiency makes it particularly suitable for deployment in resource-constrained environments, where access to advanced computational infrastructure may be limited. This work could reduce the diagnostic burden in malaria-endemic regions, streamline laboratory workflows, and minimize human error in detecting and classifying malaria parasites.

Future work will refine the system to automate all diagnostic steps, enabling more accurate and scalable malaria diagnosis in low-resource settings. This includes integrating smartphone imaging technologies to enhance accessibility in remote areas, expanding the dataset to cover additional Plasmodium species, and improving generalizability across varying staining protocols to further broaden the system’s applicability.

## Figures and Tables

**Table 1:** Data partitioning.

	Good Quality Images	Bad Quality Images	Leukocytes	Parasites
Training	151	549	9286	4972
Validation	30	120	2020	673
Testing	36	114	1315	543
Total	217	783	12,621	6188

**Table 2:** Results of the quality analysis performance.

Kernel	Quality	Precision	Recall	F1
Lineal	Bad	91%	91%	91%
	Good	91%	91%	91%
Quadratic	Bad	94%	94%	94%
	Good	94%	94%	94%
Cubic	Bad	94%	97%	96%
	Good	97%	94%	95%
Gaussian	Bad	89%	94%	91%
	Good	94%	88%	91%

**Table 3:** Detection leukocytes mask (experimental design validation).

Treatment	Color Space	Filtering	Contrast Enhancement	Precision (%)	Recall (%)	F1-Score (%)
1	Blue	no filter	CLAHE	87.52%	93.09%	89.3%
2	Saturation	no filter	CLAHE	85.28%	94.42%	88.57%
3	Gray	no filter	CLAHE	83.14%	95.71%	87.93%
4	Red	no filter	CLAHE	83.24%	95.55%	87.81%

**Table 4:** Best results for the gray, RGB, and SV scales.

Color Scale	Feature Groups	Algorithm	Accuracy (%)	Precision (%)	Recall (%)	Specificity (%)	F1-Score (%)
Gray	GLCM 72	SVM	89.00%	88.68%	88.82%	89.17%	88.75%
Green	GLCM 72	SVM	90.06%	90.06%	89.52%	90.56%	89.79%
Saturation	GLCM 72	SVM	89.92%	89.36%	88.92%	89.95%	88.66%

**Table 5:** Confusion matrix CNN model on a patch level.

	Predicted Negative	Predicted Positive
**Non-parasites**	95.6%	0.9%
**Parasites**	0.5%	3.1%

**Table 6:** Performance for SVM on different kernels.

	F1-Score		
Classifier	Trophozoite	Schizont	Gametocyte
Linear	70%	70%	55%
Quadratic	54%	58%	48%
Cube	58%	51%	52%

**Table 7:** Experiments for multiple CNN configurations.

Classifier	F1-Score			Accuracy
	Trophozoite	Schizont	Gametocyte	
1	89%	83%	73%	81%
2	87%	81%	75%	80%
3	81%	79%	78%	79%
4	87%	76%	74%	78%

**Table 8:** CNN configurations vary from hyperparameters.

			F1-Score			Accuracy
Batch Size	Epochs	Learning Rate	Trophozoite	Schizont	Gametocyte	
70	100	0.00001	85%	88%	83%	86%
80	150	0.001	84%	76%	71%	76%
50	100	0.001	85%	82%	76%	80%
50	50	0.00001	92%	84%	74%	84%

**Table 9:** Transfer learning performance.

Classifier	F1-Score			Accuracy
	Trophozoite	Schizont	Gametocyte	
Mobile Net	64%	75%	74%	72%
InceptionResNetV2	82%	74%	75%	76%
ResNet50	64%	75%	74%	72%
VGG16	82%	74%	75%	76%

## Data Availability

The dataset is available at: https://zenodo.org/record/7484518#.Y6zAr3bMLrc (accessed on 23 December 2022).
